# Decoding the view expectation during learned maze navigation from human fronto-parietal network

**DOI:** 10.1038/srep17648

**Published:** 2015-12-03

**Authors:** Yumi Shikauchi, Shin Ishii

**Affiliations:** 1 Graduate School of Informatics, Kyoto University, Kyoto 606-8501, Japan; 2 ATR Cognitive Mechanisms Laboratories, Kyoto 619-0288, Japan

## Abstract

Humans use external cues and prior knowledge about the environment to monitor their positions during spatial navigation. View expectation is essential for correlating scene views with a cognitive map. To determine how the brain performs view expectation during spatial navigation, we applied a multiple parallel decoding technique to functional magnetic resonance imaging (fMRI) when human participants performed scene choice tasks in learned maze navigation environments. We decoded participants’ view expectation from fMRI signals in parietal and medial prefrontal cortices, whereas activity patterns in occipital cortex represented various types of external cues. The decoder’s output reflected participants’ expectations even when they were wrong, corresponding to subjective beliefs opposed to objective reality. Thus, view expectation is subjectively represented in human brain, and the fronto-parietal network is involved in integrating external cues and prior knowledge during spatial navigation.

The brain creates prediction of future events based on beliefs of the environment: dopaminergic neurons in primates are activated by the reward-predicting stimulus after stimulus-reward conditioning^1^, and hippocampal place cells in rats encode potential future paths during spatial navigation^2^,^3^. The Bayesian brain hypothesis has proven success in computational modeling for perception and sensorimotor control^4^. Current beliefs generate predictions, against which sensory samples are tested to update the beliefs^5^. Although Bayesian statistics describe how a new observation and previous knowledge termed ‘prior’ can be optimally combined computationally into behaviors^6^,^7^,^8^,^9^,^10^,^11^, it has not allowed us to know on how we relate external cues to the beliefs maintained in the brain.

Here, we focus on the view expectation as an example of everyday prediction. The view expectation is the intermediary connection between an upcoming scene view and our empirical knowledge of the environment (cognitive map) during spatial navigation in a familiar context. Specifically, our fields of view are restricted to those surrounding the current location of us (Fig. 1a). Then, we predict forward-looking vision beyond the current scene view along with our moves (Fig. 1b). Such anticipation is obtained by integrating the current observation and the retained cognitive map into a comparable form to the upcoming scene view. Because this view expectation is dependent on our prior knowledge, it is subjective rather than the solely objective information provided by the environment. Thus, we tested whether subjective view expectation could be detected from human brain activities. In this study, human participants were passively navigated with observing a sequence of scene views, which were depicted in terms of three-dimensional (3D) wire-frames, accompanied by the next move direction. Then, they were asked to choose a forthcoming scene from two options (two force choice task) while being imaged by functional magnetic resonance imaging (fMRI) (Fig. 2). If participants get familiar with this environment, they can predict and choose the next scene accurately. To determine when and where neural representation for the expectation of the forthcoming scene appears, we applied a multiple parallel decoding analysis, attempting to read out different navigation-related types of information including the expectation of the forthcoming scene from six different regions in the brain (Fig. 3): medial and dorsal sites of the superior frontal gyri (mPFC and dPFC), presuneus, superior parietal cortex (sPC), hippocampal-parahippocampal cortex (HC-paraHC) and occipital cortex (OC). The medial prefrontal and posterior parietal cortices are concerned as possible sources of expectation in terms of both of rewards and potential future paths^12^,^13^,^14^,^15^,^16^,^17^,^18^. In addition, the superior frontal sulcus (included in dPFC) is thought to be specialized for spatial working memory^19^.

Based on the multiple parallel decoding, we report three main results. First, the medial prefrontal cortex (mPFC), the precuneus, posterior parietal region and occipital cortex (OC) have information on the context of upcoming scene views. Second, OC activity patterns represent various types of external cues, whereas mPFC and the parietal regions are associated specifically with view expectation. Third, the activity patterns in the parietal regions reflect participants’ expectation even when it is wrong, thus corresponding to subjective belief as opposed to objective reality. Furthermore, we demonstrate visualization of individuals’ cognitive map based on outputs of the expectation decoders. Taken together, these results suggest that view expectation is represented in the human brain as identified by fMRI voxels, and that the fronto-parietal network is crucially involved in subjective integration of environmental cues and retained cognitive map.

## Results

### Behavioral task performance

**Figure 2:**
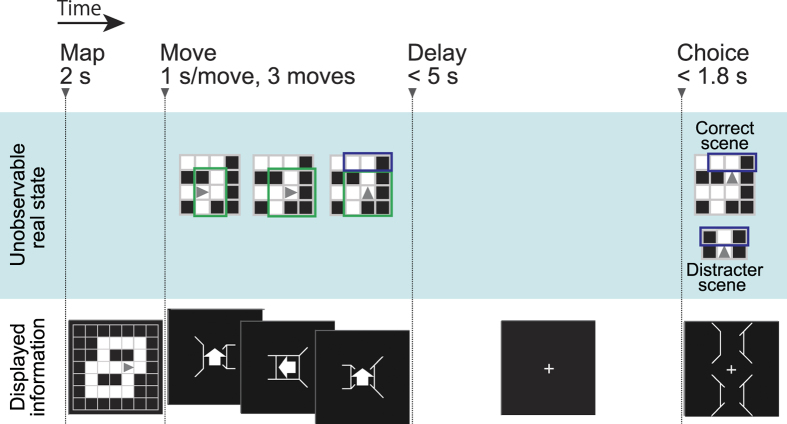
An example scene choice trial. The bottom display series show an example of presented stimuli during a single scene choice trial. In the first display, one of the three 2D maps (see Behavioral task) and a triangle indicating the initial state, position and body orientation, of the participant on the 2D map were presented (map period). The participants’ state was then moved automatically by three steps; each movement was shown by an arrow placed at the center of a 3D scene of the current state (move period). Each 3D scene showed the path/wall status of left, right, forward-left, forward-center and forward right squares of the current state (a triangle in the top ‘real state’ display). An up arrow made the participant to move forward with the same orientation, while a left (right) arrow made the participant’s orientation to turn toward the left (right) on the same square. The second display showed the 3D scene of the initial state and first movement of the participant; in this case, the participant was moved forward to the east. The participants’ orientation was changed to the north (turn left, second movement) in the third display, while the participant was moved forward (third movement) in the fourth display. The right display series show the real states of the participant, although they were hidden for the participant. Before the third movement but after presenting a preview of the third move, there was a delay period during which only a fixation point was presented (the left fifth display), then the participant was requested to choose the upcoming scene from two options: a correct scene to be seen after the movement and a distracter scene (the sixth display) (choice period). Because the third movement was fixed to be forward (the fourth display), the prediction of the upcoming scene after the third movement always constituted three new view parts, whose direct information was not seen during the move period. The distracter scene was one of the three view parts flipped (the forward-left view was turned from a path to a wall in this example).

**Figure 3:**
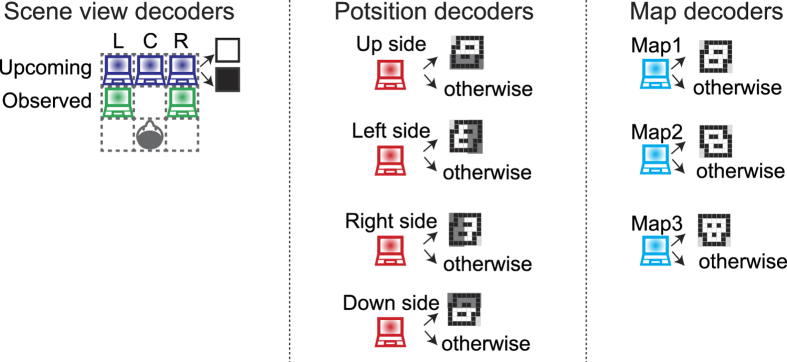
Analysis overview. Four types of navigation-related decoder were applied to the brain activity during the scene choice task. The scene view decoders were of three binary classifiers in terms of upcoming square: forward-left, forward-center and forward-right, and two binary classifiers in terms of observed square: left and right. The position and map decoders were for the rough position and the map, respectively, where the participant stayed in the current trial. In this example’s case, the up-side, right-side and map 1 classifiers’ output should be positive because the current location was upper right on map 1.

**Figure 4:**
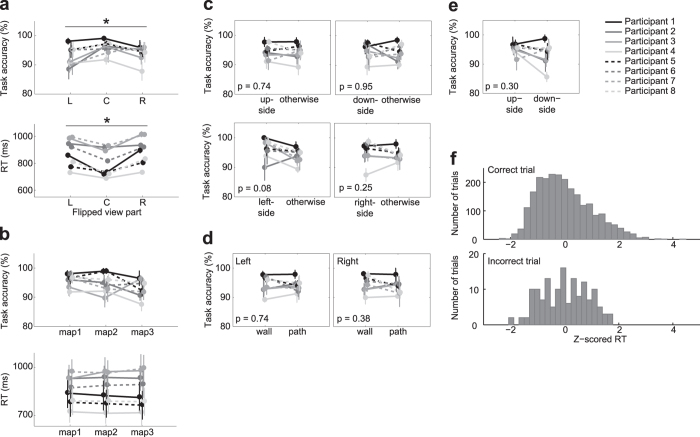
Behavioral results. They are shown in terms of task accuracy (how accurately the participants performed the scene choice task) and reaction time (RT), both averaged over sessions. Error bars indicate SEM across sessions (n = 5). (**a**) In the forward-center (C) trials, the task accuracy was higher and the RT was shorter than in the forward-left (L) and forward-right (R) trials (Friedman-test, *p* < 0.05). (**b**) No significant difference in the task accuracy or RT was found between three maps (Friedman-test). (**c**) The position of the participants after the third movement did not significantly affect the task accuracy (Wilcoxon signed rank test). (**d**) The task accuracy did not show significant difference between the statuses (wall/path) of both of the left and right sides of the observed scene view (Wilcoxon signed rank test). (**e**) The position, up or down, showing the correct scene in the choice period did not significantly affect the task accuracy (Wilcoxon signed rank test). (**f**) Histograms of RTs in correct trials (top panel) and in incorrect trials (bottom panel). Each RT was converted to z score per session.

Eight participants performed the scene choice task that was a modified version of the pre-learned maze navigation game presented previously^20^; the participants were requested to choose as soon as possible between two options represented by 3D wire-frame scenes, a correct upcoming scene and a distracter scene (Fig. 2). The options appeared after the participants’ state was moved automatically in one of three two-dimensional (2D) pre-learned mazes by three steps and before seeing the scene after the third movement. The scene choice task required the participants to identify small differences between the two options with only one of three view parts, forward-left (L), forward-center (C) or forward-right (R) flipped (Figs 2 and 3, Behavioral task). Although an allotted time was limited (choice period, 1.8 s) and any information about the scene choice task was not presented until the choice period, the participants showed satisfactorily high performance (mean ± SD): 93.4 ± 2.6% correct, 5.6 ± 2.3% incorrect and 1.0 ± 1.0% missed. Missed trials, in which the participants could not press an answering button in the allotted time, were excluded from the following analyses. To answer correctly and rapidly, the participants were required to predict the upcoming scene, and the behavioral result above showed that participants predicted the upcoming scene well. A Friedman test for participants’ task accuracy and reaction time (RT) demonstrated significant differences between flipped view parts (Fig. 4a; task accuracy, χ^2^(2, *N* = 40) = 33.7, p < 0.05; RT, χ^2^(2, *N* = 40) = 9.2, *p* < 0.05); in the forward-center flipped trials, the participants had a tendency toward higher accuracy and shorter RT than in other trials. By contrast, there were no apparently significant differences in the task accuracy and RT between the three maps (Fig. 4b; task accuracy χ^2^(2,*N* = 40) = 5.9, *p* = 0.05, χ^2^(2,N = 40) = 0.1, *p* = 0.95). Additionally, the task accuracy did not show significant differences between the rough position on the maze (Fig. 4c; Wilcoxon signed rank test, up-side *vs.* otherwise: *p* = 0.74; down-side *vs.* otherwise: *p* = 0.95; left-side *vs.* otherwise: *p* = 0.08; right-side *vs.* otherwise: *p* = 0.25), between the statuses (wall/path) of the current observable scene (Fig. 4d; Wilcoxon signed rank test, left: *p* = 0.74, right: *p* = 0.38) or between the positions (up/down, see Fig. 3) of the target option (correct scene) (Fig. 4e; Wilcoxon signed rank test, up-side *vs.* down-side: *p* = 0.30). In addition, there was no significant difference in the RT’s distribution between correct trials and incorrect trials (Fig. 4f; Wilcoxon rank sum test, *p* = 0.45); although the RT would reflect the difference in cognitive processes between view parts, it has no information about which option was chosen by the participants in each trial.

### Time-course decoding analysis

**Figure 5:**
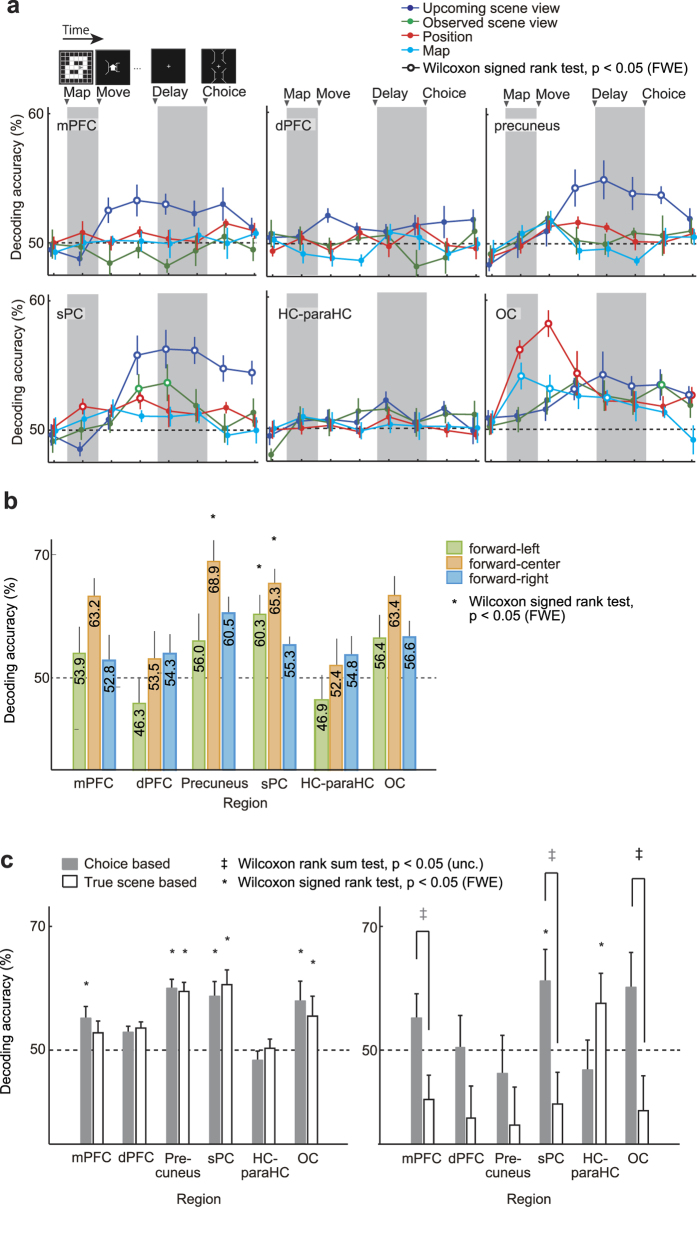
Decoding results. Error bars indicate SEM across participants (n = 8). (**a**) Time-course decoding analysis along the scene choice task. Each panel shows the decoding accuracy from one of the six ROIs, which was evaluated by the leave-one-session-out (LOSO) procedure. At each event timing, fMRI signals during a single scan (2 s) just after that timing in the time-course were used for decoding. Open circle indicates significantly higher accuracy than the chance level. (**b**) The decoding accuracy was evaluated when the trials were separated into three categories (forward-left, forward-center and forward-right), depending on the target (the pair of the correct view and the distracter view) in the scene choice task; the decoding accuracy was evaluated by the LOTO procedure. (**c**) The decoding accuracy in correct (left) and incorrect (right) trials was evaluated by the decoders that trained with incorrect trials. We used two methods: one involved decoding the distracter’s view as right as the participants actually chose it, while the other involved decoding the true view as right as it should be ideally chosen. The decoding accuracy was evaluated by the LOTO procedure. Double daggers indicate significant difference in the accuracy between these labeling methods (p values evaluated by the Wilcoxon rank sum test).

To determine when and in what region the neural bases were recruited in the scene choice trials, we performed voxel-wise fMRI analysis along the whole task. We constructed four sets of binary decoders (Fig. 3, comparison between status-specific decoders): three upcoming scene view decoders, two decoders for observed scene view in the third move, four decoders of position in the third move, and three map decoders. Each decoder associated voxel-wise activity patterns in each of six anatomical regions of interests (ROIs): mPFC, dPFC, precuneus, superior parietal cortex (sPC), HC-paraHC and OC (Table 1, Materials and Methods). FMRI data preprocessing included trend removal, noise reduction and temporal normalization (see Materials and Methods). Here, any hemodynamic response function was not convolved to avoid confounding any prospective activation related to the behaviors (option choices) induced by visual presentation in the subsequent choice period. When we used trials in which the participants answered correctly in the scene choice task (correct trials) for both of decoders’ training and their evaluation, we found that decoding of the upcoming scene view was possible from the single-scan (2 s) activities in the mPFC, precuneus, sPC and OC (Fig. 5a). Interestingly, the decoding accuracy from those regions was high especially in the delay period, and was even better in the choice period. Activities in the sPC allowed us to decode the observed scene view in addition to the upcoming scene view: for the fourth scan (move period), 53.2%, *p* < 0.05 (with Bonferroni correction for comparing multiple time points and decoders); for the fifth scan (early delay period), 53.7%, *p* < 0.05 (Bonferroni-corrected). Most notably, activities in OC allowed us to decode the position in addition to the upcoming scene view: for the second scan (map period), 56.2%, *p* < 0.05 (Bonferroni-corrected); for the third scan (move period) 58.2%, *p* < 0.05 (Bonferroni-corrected); for the fourth scan (move period), 54.3%, *p* < 0.05 (Bonferroni-corrected); for the eighth scan (choice period), 52.7%, *p* < 0.05 (Bonferroni-corrected). However, there were no significantly decodable scan timings when using dPFC or HC-paraHC activities. These decoding results remained unchanged even if we used the activities in HC or paraHC alone.

### Upcoming scene view decoding from delay-period brain activity

To study responses of brain activities to the view expectation in terms of view parts, we evaluated the three upcoming-view-part-dependent decoders with correct trials. In each trial of the scene choice task, the participant’s choice from two options allowed us to know what had been the participant’s expected scene for a single view part (corresponding to the flipped view part in the distracter). To construct effective decoders that exploited this character, we divided the fMRI data samples into three groups in terms of the flipped view parts, so that three view-part-dependent decoders (forward-left, forward-center and forward-right) were individually trained based on the three groups. Their performance was examined by the leave-one-trial-out (LOTO) procedure (Evaluation of the decoders for the upcoming scene view), with Bonferroni correction to deal with multiple anatomical ROIs. Based on the time course analysis above, we used the fifth scan (early delay period) and the sixth scan (just before the choice period); i.e., when the participants were preparing to see the options to be chosen (Fig. 2).

When using sPC activity, the decoders allowed us to read out two of the three view parts in the upcoming scene: Wilcoxon signed rank test, forward-left: 60.3%, *p* < 0.05 (Bonferroni-corrected); forward-center: 65.3%, *p* < 0.05 (Bonferroni-corrected); forward-right: 55.3%, *p* = 0.31 (Bonferroni-corrected) (Fig. 5b). By contrast, a high performance was obtained for only forward-center views from the precuneus activity.

To further assess whether these decoders reflect the participants’ mental state or some tendency of the viewed stimuli, we used samples when the participants answered incorrectly (incorrect trials). When evaluating the decoding accuracy for incorrect trials, we used two different schemes: (1) decoding the distracter’s view was regarded as correct because the participants actually chose it, and (2) decoding the true view was regarded as correct because it should be ideally chosen. Each of the three view-part-dependent decoders was trained using samples from both of the correct and incorrect trials and evaluated in terms of the LOTO cross-validation (CV) performance for the incorrect trials. Consistent with the time-course analysis (Fig. 5a), in the mPFC, precuneus, sPC and OC, both of the decoders trained with incorrect trials achieved significantly high correct rates on the correct trials (Fig. 5c left). Interestingly, in the mPFC, sPC and OC, the averaged decoding accuracy when regarding the distracter’s view as right was significantly higher than that for answering the true view (Wilcoxon rank sum test, *p* < 0.05) (Fig. 5c right). This result supports that participants’ brain activity patterns included subjective expectations of the upcoming scene view.

### Map visualization

**Figure 6:**
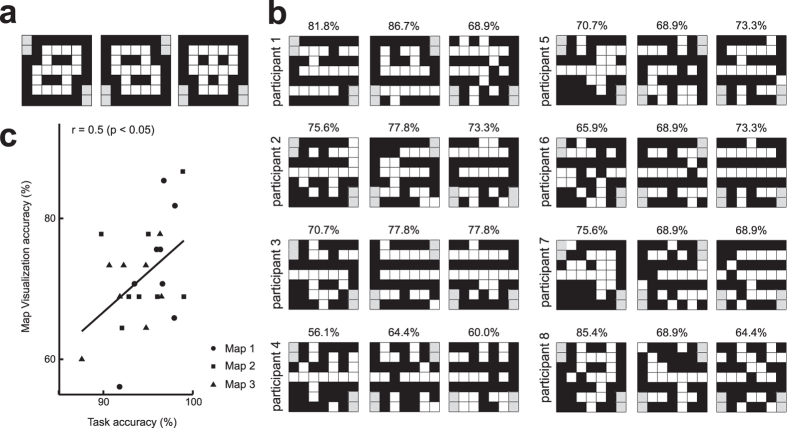
Real maps and visualized maps based on decoding of view expectation from fMRI activity patterns. (**a**) Three 7 × 7 maps used in the experiment. (**b**) Each square of the visualized map denotes the average of the outputs from the three upcoming-scene-view-dependent decoders (forward-left, forward-center and forward-right) as white (black) if the averaged value was larger (smaller) than a threshold, although the number of samples in which the corresponding square was the target of prediction varied depending on the squares. When visualizing a specific map, fMRI data and the true labels (path/wall status) in the trials where the map was used in the scene choice task were never used (the LOMO procedure), and an output from each decoder was an analogue value ranging between 0 and 1, showing the path probability (0, less path-like; 1, more path-like). Because this subjective probability can be biased because of the number unbalance between paths and walls, the threshold to binarize was set to select the same number of paths as in the real map. Gray squares were excluded from the evaluation. At the top of each visualized map, the visualization accuracy for the squares in the map is displayed. (**c**) Scatter plot depicts a significant relationship between the behavioral task accuracy of individual participants and their map visualization accuracy. For each participant, there are three points corresponding to the three maps.

To remove the decoder’s dependency on map topography, we performed another kind of CV, leave-one-map-out (LOMO), in which each upcoming-view-part-based decoder did not use the trials when the participants were on a specific map when training, but was evaluated by those trials. Thus, each decoder could not use the topographic information of the map. This LOMO CV also allowed us to visualize the map by simply arranging the scene view predicted by the upcoming-view-part-based decoders based on the participants’ fMRI signals just before seeing that view part, as a 2D representation of the prediction of the map (Fig. 6b, 2D arrangement of decoded wall-status). Because most squares in the visualized map show the average of outputs from multiple view-part-based decoders and multiple trials, the square-wise decoding accuracy (map visualization accuracy) of the whole map (mean map visualization accuracy: map 1: 72.7 ± 9.2%; map 2: 72.8 ± 7.3%; map 3: 70.0 ± 5.7; SD is over eight participants) was higher than those of view-part-dependent decoders (Fig. 5b).

The most far-reaching question we posed was whether this decoded view expectation would correlate with the participants’ choice behavior. As shown in Fig. 6c, we found that the square-wise decoding accuracy was significantly correlated with individuals’ performance of the scene choice task.

## Materials and Methods

### Participants

Eight healthy participants (1 female author, 7 male; aged 21–28 years) took part in this experiment. Each participant gave written informed consent, and all experiments were approved by the Ethics Committee of the Advanced Telecommunications Research Institute International, Japan. All methods were carried out in accordance to the approved guidelines. We verified that the main decoding results remained unchanged even if the author was removed from the participant set by a post-hoc analysis.

### Behavioral task

The experimental task (Fig. 2) consisted of 320 trials, spread out over five sessions (64 per session). The sessions were separated by breaks of a few minutes each. Each trial consisted of four periods. In the first period (map period), participants were presented with an initial position and orientation on one of three 2D maps (2 s). The maps had 7 × 7 squares of white paths and black walls with no dead ends, cross roads, white patches (2 × 2 squares consisting only of paths), black patches or checker patterns (Fig. 6a). The borders of the three maps were all walls. The topographies of the three maps were symmetrical and simple so that it was easy for the participants to memorize the topographies but still hard to identify their position from only a single 3D scene. A 3D scene in wire-frame form of the wall status (path/wall) of the left, right, forward-left, forward-center and forward-right squares of the current state was presented (i.e., was partially observable). During the second period (move period), each participant’s state was moved automatically by three steps (1 s/move). For each movement, after being presented with a white arrow on the current scene view giving a preview of the next move, a new 3D scene with a new preview was presented. The first and second movements were one of three movement types (move forward or turn left/right), while the third movement was fixed to be forward. Before the third movement but after presenting a preview of the third move, there was a delay period (5 s, 60 trials/session. The delay period was either 1 s, 3 s or 5 s in each trial, to introduce ‘jitter’ to the raise in the brain activity; the order of this jitter time was pseudo-randomized across trials. In the following analyses, we only used the trials, 60 trials/session, whose delay time was 5 s). After the delay period, the participants were then requested to choose as soon as possible between two options, a correct scene to be seen after the third movement and a distracter scene, both represented by 3D wire-frame scenes, using an MRI compatible button box (choice period, < 1.8 s). The new 3D scene after the third forward movement contained three newly observed view parts (forward-left, forward-center and forward-right), which had never been seen in the preceding move period or in the delay period. A distracter scene was one of the upcoming three view parts flipped without producing a new dead end, cross road, white/black patch or checker pattern, where flipping was balanced between the upcoming three view parts, so the participants could not predict the scene choice task. After the participants made a choice (or if they failed to answer within 1.8 s), the options were replaced immediately by blank and the next trial started. After each session, the rate of correct answers in the session was presented to the participants. We prepared sets of 2D maps, initial states, three movements and distracters, based on diversity, so that every movement was effective (e.g., there was no wall in the forward direction after the second movement) and there was no common tendency to the participants’ state after the three movements; the rate of wall/path in the scene choice task (mean ± SD) was approximately 50% for all three view parts: forward-left 0.49 ± 0.01, forward-center 0.50 ± 0,01 and forward-right 0.50 ± 0.02. The SD was over five sessions. According to these criteria, 167 sets were prepared and used across the experiments. These sets were common for all the participants, but their order was randomized within a session such to be counterbalanced across participants. Here, we prioritized the unpredictability of the scene choice task, while the scene occurrence was not uniform, reflecting the map topography; some sets were presented more than once (max 5, mean 1.95).

On the day before scanning, participants were given verbal and written explanations of the aim and procedures of the experiment, and practiced two types of training sessions outside the MRI scanner. In the first training session, the participants performed a free navigation task in another map with larger 9 × 9 squares, in which they selected their movements by their own, so as to learn the relationship between 2D topography and 3D scenes. Participants were presented with both the current state on a 2D map and the 3D scene of the current state. After the participants reported that they were familiar with the 2D-3D association, in the second type of training they performed sessions of a scene choice task, which was the same as the main experimental task that they would take part in the subsequent day. This second type of training was ended after the participants could choose the true upcoming scene with more than 80% accuracy.

### Data acquisition and preprocessing

A 3.0-Tesla Siemens MAGNETOM Trio A Tim scanner was used to acquire interleaved T2*-weighted echo-planar images (EPI) (TR = 2 s, TE = 30 ms, flip angle = 80°, matrix 64 × 64, field of view 192 × 192, voxel 3 × 3 × 4 mm, number of slices 30). A high-resolution T1 image of the whole head was also acquired (TR = 2250 ms, TE = 3.06 ms, flip angle = 90°, field of view 256 × 256, voxel 1 × 1 × 1 mm).

The first six scans of each run were discarded so as not to be affected by initial field inhomogeneity. The acquired fMRI data underwent 3D motion correction using SPM8 (http://www.fil.ion.ucl.ac.uk/spm). The data were coregistered to the whole-head high-resolution anatomical image, and then spatially smoothed with a Gaussian kernel filter (FWHM, 8 mm). In a post-hoc analysis, we confirmed that a smaller (3 mm) setting of the spatial smoothing did not change any of our conclusions.

### Regions of interest

During spatial navigation of rats, firing patterns of hippocampal system was reported to represent spatial status^21^,^22^. Neurons in medial prefrontal cortex (mPFC) and posterior parietal regions showed choice- and proceeding- specific firing patterns^23^,^24^. Early visual areas in the occipital cortex (OC) retain specific information about contents of visual working memory when no physical visual cue is present^25^. Moreover, spatial working memory is thought to be represented in dPFC^19^. Based on these previous studies related to the view expectation, we examined six bilateral regions of interest (ROIs) in the decoding analysis: the mPFC, dPFC, precuneus, superior parietal cortex (sPC), hippocampal-parahippocampal cortex (HC-paraHC) and OC (Table 1). We integrated the HC and paraHC into a single region, making the size of the combined region (HC-paraHC) comparable to those of the other ROIs. Using ITK-SNAP (www.itksnap.org)^26^, the six anatomical ROIs were identified on the high-resolution T1 image of each participant in reference to the Automated Anatomical Labeling^27^, whose mean voxel numbers and their variances over the eight participants are summarized in Table 1. The fMRI signals from these ROIs then underwent quadratic polynomial trend removal and noise reduction by means of singular value decomposition (K = 3), and were then normalized within each session.

### Labeling of fMRI data

Trial-based fMRI signals taken in the scene choice task were labeled as positive or negative depending on four types of status in the scene choice task: an upcoming scene view, an observed scene view in the third move, a position in the third move, and a map. The label for the upcoming scene view was set based on the status, path (positive, or 1) or wall (negative, or 0), associated with the three view parts of the scene choice task; i.e., forward-left, forward-center and forward-right (Fig. 3). Although the participants were assumed to predict the next scene but could not predict the distracter, the delay period fMRI signals involved activity related to the prediction of the upcoming three view parts. To incorporate the view part dependency obtained by the behavioral analysis (Fig. 4) and to avoid overly complicated procedures in the scanning experiment, the distracters were set as one-view-part flipped ones; this setting was appropriate for the construction of binary classifiers (decoders). Similarly, the label for the observed scene views consisted of two codes; whether the left and right sides at the state after the third movement were path (positive, or 1) or wall (negative, or 0), which are observable as the forward-left and forward-right view parts before the third movement. For the position, the label consisted of four binary codes, as follows: whether the position after the third movement was located in the upper side (positive, or 1) or not (negative, or 0), in the lower side or not, in the left side or not, and in the right side or not. Because each of the three maps had 7 × 7 squares, the codes could be positive both for the upper (left) side and lower (right) side when the position was at the middle row (column) of the map. The map label consisted of three binary codes, each of which signified each of the three maps 1, 2 and 3; e.g., (1, 0, 0) indicates map 1.

### Decoding analysis overview

Multiple parallel decoding analysis was performed on single trials based on the fMRI signals of the voxels belonging to the six ROIs above. Each binary decoder was trained in a supervised fashion to associate the normalized voxel signals (input variables) with the above-mentioned labels (output variables) in the scene choice task. For each binary classifier, we used a linear classifier, sparse logistic regression (SLR)^28^. Although the number of inputs was as large as 200–1000 (Table 1), hierarchical Bayesian setting called automatic relevance determination and its variational Bayesian approximation can ignore input variables irrelevant to the classification; such sparseness would be effective in increasing the generalization capability. In our implementation, default values in the SLR toolbox were used for all parameters of the classifiers.

### Comparison between status-specific decoders

For each participant, 12 binary SLR classifiers (three decoders for the upcoming scene view, two for the observed scene view, four for the position and three for the map), termed status-specific decoders in the multiple parallel decoding, were trained individually (Fig. 3). 12 binary status-specific decoders were trained for each scan and for each of the six ROIs over the single trials, and the total 72 (12 × 6) decoders were evaluated. When evaluating the trained status-specific decoders, we used a leave-one-session-out (LOSO) cross-validation (CV). In the LOSO procedure, each decoder was trained using the training data set from which one session was removed, and the removed session was used to validate the trained decoder. By changing the removed session one by one, we could evaluate the CV performance of the decoders. When reporting the overall decoding accuracy of each status-specific decoder (Fig. 5a), the mean accuracy averaged over the constituent code-dependent decoders (e.g., forward-left, forward-center and forward-right, for the upcoming-scene-view-dependent decoders) was used.

For each decoder, samples with the positive and negative labels in the original training data set were unbalanced (The ratio (and the SD over eight participants) of positively-labeled samples in the set of correct trials was as follows: the upcoming scene view, forward-left 0.28 (0.01), forward-center 0.67 (0.02) and forward-right 0.28 (0.01); the observed scene view, left 0.35 (0.02) and right 0.36 (0.01); the position, upper side 0.81 (0.03), lower side 0.68 (0.01), left side 0.59 (0.01) and right side 0.63 (0.01); and the map, map1 0.28 (0.01), map2 0.31 (0.02) and map3 0.41 (0.03)). Because simple use of such an unbalanced data set to train binary decoders may introduce a bias to the decoders, positive/negative samples in each training data set were resampled to remove the unbalance. Let *N*_pos_ and *N*_neg_ be the numbers of positive and negative samples in the original training data set, respectively. If *N*_neg_ > *N*_pos_, then *N*_pos_ negative samples were randomly subsampled, otherwise *N*_neg_ positive samples were subsampled, so that the numbers of positive and negative samples became equal. An output from each SLR decoder was an analogue value, ranging between 0 and 1, implying the probability of the label being one (positive). When evaluating the decoder’s accuracy, we binarized the analogue value by applying a threshold value of 0.5.

### Evaluation of the decoders for the upcoming scene view

When evaluating each of the three decoders for the upcoming scene view, the fMRI data samples for each participant were divided into three groups in terms of the flipped view parts in the scene choice task. For each participant and for each of the six ROIs, three binary SLR classifiers were trained individually. The set of voxel signals averaged over two scans (2 × TR = 4 s) just before presenting the choice options (the choice period) was used as a sample for the decoders. The three decoders were then constructed based on the three corresponding separated data samples. Thus, the label unbalance in the training data set was minimal (prior chance levels of the decoder after removing miss trials, forward-left: 51.9 ± 2.2%, forward-center: 50.9 ± 0.5%, forward-right: 51.4 ± 1.3%). When evaluating the three upcoming scene decoders, we used a simple leave-one-trial-out (LOTO) procedure, in which each decoder was trained by the training data set from which one trial was removed, and the removed trial was used for validation (Fig. 5b,c).

### 2D arrangement of decoded wall-status

When visualizing the maps (Fig. 6b), we used an alternative validation method termed the leave-one-map-out (LOMO) procedure. Each decoder for a single view part (either of forward-left, forward-center or forward-right) was trained using the data set in the scene choice task of the corresponding view part, but the training trials were restricted to those using two of the three maps. The trials employing the remaining map were kept for validation. The objective of the LOMO procedure was to examine the generalization capability of the decoder’s neural basis by removing the dependency on specific maps. In the visualization, we calculated the square-wise value (corresponding to the probability of path of that square), 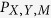, as the mean of the decoders’ analogue outputs 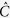 with the sigmoidal rule:


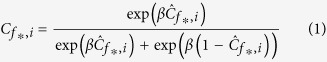



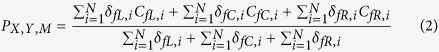



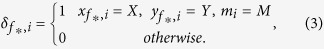


where β is an inverse-temperature parameter (β = 5), 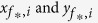
represent the horizontal and vertical coordinates, respectively, of the target square of the decoder either of forward-left, forward-center or forward-right in the *i*-th trial and 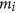 is the map index of the trial *i*. *N* is the number of trials in which the map *M* was used by the participant. The averaged numbers of the superposition 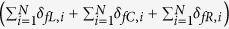 were 6.1 ± 6.8 for the squares on map 1, 6.5 ± 5.9 for those on map 2 and 8.7 ± 4.0 for those on map 3 (the SD is over squares). We applied a map-dependent threshold (see legend of Fig. 6b) to the analogue value above to make the visualized map a binary one.

## Discussion

### Status-specific decoding analyses

Our decoding analyses found that the parietal regions (precuneus and sPC) represented the prediction of upcoming scene view in the scene choice task (Fig. 5), implying that they are involved in navigation in partially observable navigation environments. We found that various kinds of navigation-related information (i.e., the observed scene view, the map and the position), as well as the upcoming scene view, were decoded from the OC in the delay period, suggesting that the visual system in the OC is used for prediction accompanied by collecting information from observations. These results are consistent with previous decoding studies on visual working memory, mental rotation and perceptual speed^29^,^30^,^31^,^32^. Although decoding performance was poor from the hippocampal system (HC-paraHC) and dorsal part of PFC (dPFC) throughout the task, the mPFC and precuneus showed good decoding performance only for the upcoming scene view. Notably, the sPC showed the highest decoding accuracy for the upcoming scene view (Fig. 5a); when we decomposed the scene view to be predicted into three view parts (forward-left, forward-center and forward-right), decoding from the sPC showed significantly high accuracy. These results are consistent with the findings that sPC contributes to the manipulation of egocentric spatial information^33^,^34^.

The time-course decoding analysis (Fig. 5a) provided information on the processes during the scene choice task; in the move period, prior to precuneus and sPC, the upcoming scene view was represented in the mPFC. According to Yoshida and Ishii (2006)^20^, in the mPFC, multiple proposals for the current position are maintained and resolved as the information from the observations increases. Thus, brain activity in the mPFC may have contributed to retention and updating of multiple proposals, which should be preceded to the prediction of the new scene. Besides, the mPFC is considered the key nodes of the default mode network^35^. The information of the upcoming scene view expectation, which was involved in mPFC activities, may thus be used for manipulating multiple proposals within the dynamics embedded in spontaneous brain activities. The position decoders showed the highest accuracy when decoded from the OC activity in the early period of the scene choice task. Because the position identification was crucial to perform well in the scene choice task on these rather symmetrical maps, a large cognitive resource might have been allotted to position identification, especially in the move period.

Because there is little known regarding the neural bases of prediction, we introduced a decomposed decoding technique, producing three binary decoders (forward-left, forward-center and forward-right), rather than a single eight (=2^3^)-class decoder, for the upcoming scene view. A similar method used to read out complex viewed stimuli from primary visual fields was previously reported^36^. Such parallel decoding methodology shows conceptual correspondence to the encoding process by the brain, which is implemented within massively parallelized machinery.

Previous decoding studies often used localization tasks to define the functional ROIs of individual participants^31^,^37^,^38^. For higher-order cognitive functions such as view expectation, however, functional ROIs are too dependent on the localization task itself, and may not be very suitable; as higher-order cognitive functions may involve multiple sub-processes, and as fMRI signals from higher-order cortices are relatively weak, it can be difficult for the localization task to identify responsible regions that are recruited only in some but essential parts of the whole task. As such, we preferred anatomical ROIs that were parcellated based only on individuals’ anatomical information, rather than functional ROIs. For this purpose, we used a sophisticated software for anatomical parcellation^26^, which was also used in a study of memory decoding from the HC and paraHC^39^.

In the present study, we used structured maps rather than unstructured counterparts. The use of structured maps is advantageous in that they are more natural than unstructured maps, so they have a straight link toward natural navigation in real environments that should be highly structured. Note here that our view-part-dependent decoders did not use any allocentric structure information (Comparison between status-specific decoders), although the structures in the maps could provide important clues for the participants to perform not only localization but also prediction. Our time-course decoding analysis (Fig. 5a) actually showed the participants’ orchestrated processing between the view expectation in the mPFC, precuneus and sPC, and the manipulation of visual and hence structural cues in the OC.

### Decoding of expectation in decision making

Especially for decision-making in complicated environments, as for navigation in partially observable environments, the estimation of the current state and the prediction of the next state are essential sub-processes. Indeed, combining state estimation in terms of the ‘belief state’ (i.e., the posterior distribution of the current state) by means of optimal Bayesian observer and value evaluation in the space of belief states is known to produce optimal decision-making, even in partially observable environments^8^. Although we focused on the prediction of the upcoming scene rather than value evaluation, the next step would be to examine the crosstalk between the scene prediction and the prediction-based decision-making. Consistent with recent findings that functional connectivity within a fronto-parietal network is correlated with the performance of primary category cueing^40^, we found that contents in view expectation were decoded from and hence represented by fMRI activities in the mPFC, precuneus and sPC.

There are several previous reports of fMRI-voxel-based decoding in 3D navigation environments. Hassabis *et al.* (2009)^41^ decoded the position of the participants from fMRI activities in the HC and paraHC when they navigated to one of four corners in one of two 3D environments by identifying their positions based on the cues placed on the wall. Related to this study, Rodriguez demonstrated the decoding of working memory of goal direction when participants navigated to a goal position (E, W or N) based on cues placed at a specific position (S)^42^; fairly high decoding performance was obtained by using voxels in the medial prefrontal gyrus (51.4%), hippocampus (47.7%) and inferior parietal cortex (49.2%). More recently, it has been reported that the location and direction during navigation are encoded to activities in medial parietal regions^43^,^44^. In our study, on the other hand, we found no evidence to suggest that position/map information is represented in neither of the HC-paraHC, sole HC, or sole paraHC. Because the computational cost to manipulate multiple proposals and predict a plausible next scene while resolving the proposals (these are speculated to be performed in the fronto-parietal network) was high in our task, the HC and paraHC could have shown relatively low decodable activities.

Here, we mainly focused on the fronto-parietal network, which have been considered as the source of anticipation; therefore, we excluded other brain regions that could be involved in spatial information processing: the response of dorsal premotor cortex is related to spatial attention and memory^45^; the nucleus reuniens is at a passing point for spatial anticipation from the mPFC to the hippocampal system^18^. Functional dissociation of view expectation from other spatial information processing remains as one key issue to be clarified. Actually, our decoders presented sustained decodability from the delay period activity of the precuneus and sPC but not from that of the dPFC (Fig. 5a). This is in contrast to the previous work in which, the dPFC showed sustained activity during spatial information processing involved in working memory^19^.

### Choice behavior and view expectation

In contrast to the previous studies above, we focused on decoding of prediction as it reflects the intracranial operation of update of beliefs, a crucial cognitive process in decision-making in a complicated environment like the real world. If we assume that the participant’s model of maintaining and resolving multiple proposals for the current state in our partially observable environment is an optimal Bayesian observer, the prediction of the next scene corresponds to the construction of a prior belief. According to this scenario, one aim of our study was to decode prior belief in the brain. Both of the choice-based and true-scene-based decoders showed high performance for reading out the view expectation in correct trials, whereas in incorrect trials, the choice-based decoders were significantly better than the true-scene-based decoders (Fig. 5c). It is natural that the two types of decoders had no apparent difference in their accuracy for correct trials when we take into account that the number of incorrect trials is much smaller than that of correct trials. Interestingly, our decoders showed high performance for reading out the participants’ inaccurate prior belief. Thus, we could predict the participants’ decision (option choice), which would be biased by their prior belief, using fMRI signals before the options were presented. Because prior belief is the origin of creativity as well as biases and illusions, prior decoding is a first step toward understanding of such human-specific cognitive processes. Such a prior belief is largely reflective of the memory stored in the brain, and we were able to actually read out the cognitive map memorized and operated in the brain through this prior decoding (Fig. 6b). Interestingly, decoding accuracy in the map visualization (Fig. 6b) was significantly correlated with task performance by the participants (Fig. 6c). This result implies that the prior belief constructed based on the cognitive map introduced a bias and hence a mistake into the human decision-making, in particular, into the expectation of the upcoming scene view in this partially observable navigation environment.

## Additional Information

**How to cite this article**: Shikauchi, Y. and Ishii, S. Decoding the view expectation during learned maze navigation from human fronto-parietal network. *Sci. Rep.* **5**, 17648; doi: 10.1038/srep17648 (2015).

## Figures and Tables

**Figure 1:**
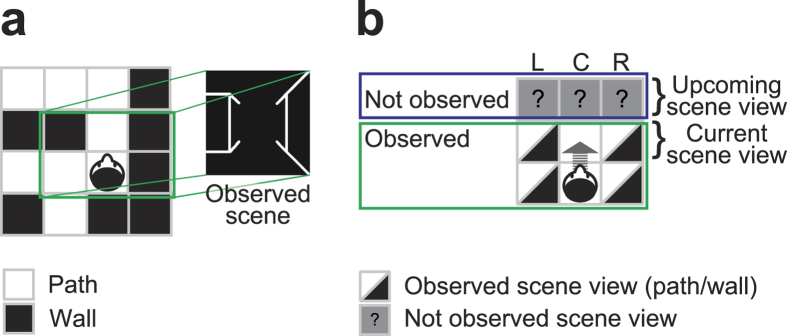
A conceptual scheme of the navigation environment. The environmental information consists of already seen and not observed spaces (squares). There are two types of squares: white paths and block walls. (**a**) An example of environments and scene view. A green box indicates a field of view for this participant. (**b**) After moving forward, here from the bottom-center square to the middle-center square in the green box, this participant observes the wall statuses of top-left, top-center and top-right squares. L, left; C, center; R, right.

**Table 1:** Datasets were created by fMRI voxels from six brain regions defined anatomically and segmented manually.

Region	Mean number of voxels	SD
Medial prefrontal cortex (mPFC)	577.1	133.2
Dorsal prefrontal cortex (dPFC)	470.6	127.2
Precuneus	644.8	40.6
Superior parietal cortex (sPC)	671.4	179.8
Hippocampal and parahippocampal cortex (HC-paraHC)	366.5	113.1
Occipital cortex (OC)	858.3	117.6

Mean number of voxels is over eight participants, and SD is the standard deviation of the voxel numbers over the participants.
